# Postoperative spinal epidural hematoma resulting in cauda equina syndrome: a case report and review of the literature

**DOI:** 10.4076/1757-1626-2-8584

**Published:** 2009-07-16

**Authors:** Tuncay Kaner, Mehdi Sasani, Tunç Oktenoglu, Bayram Cirak, Ali Fahir Ozer

**Affiliations:** 1 Department of Neurosurgery, Pendik State HospitalDr. Orhan Maltepe cd. No:17, Pendik, 34890Istanbul, Turkey; 2 Department of Neurosurgery, American HospitalGüzelbahçe sk. No:20, Nişantaş1, 34365Istanbul, Turkey; 3 Department of Neurosurgery, Pamukkale University School of MedicineBursa cd. No:119, Kinikli, 20070Denizli, Turkey

## Abstract

Spinal epidural hematoma is a well known complication of spinal surgery. Clinically insignificant small epidural hematomas develop in most spinal surgeries following laminectomy. However, the incidence of clinically significant postoperative spinal epidural hematomas that result in neurological deficits is extremely rare. In this report, we present a 33-year-old female patient whose spinal surgery resulted in postoperative spinal epidural hematoma. She was diagnosed with lumbar disc disease and underwent hemipartial lumbar laminectomy and discectomy. After twelve hours postoperation, her neurologic status deteriorated and cauda equina syndrome with acute spinal epidural hematoma was identified. She was immediately treated with surgical decompression and evacuation of the hematoma. The incidence of epidural hematoma after spinal surgery is rare, but very serious complication. Spinal epidural hematomas can cause significant spinal cord and cauda equina compression, requiring surgical intervention. Once diagnosed, the patient should immediately undergo emergency surgical exploration and evacuation of the hematoma.

## Introduction

Spinal epidural hematoma (SEH) is an uncommon cause of acute cauda equina syndrome [1-4]. Asymptomatic small epidural hematoma almost always occurs following spinal surgeries that involve laminectomy [5-8]. The incidence of postoperative SEHs that necessitate surgical intervention because of neurological deficits, such as clinically significant spinal cord or nerve root compression, is extremely rare [1-4]. The first case of SEH was described and reported by Jackson in 1869 [1,4]. Spinal epidural hematoma could be a result of coagulopathy, trauma, vascular anomalies, spontaneous reasons, anticoagulation therapy, spinal catheterization, and (rarely) spinal surgery [1,2,4,9-13]. In the literature, incidence rates of postoperative SEHs requiring surgical evacuation range from 0.1 % to 0.2% [1-4]. Although rare, postoperative SEHs that necessitate surgical intervention by causing devastating neurological deficits can be a significant cause of morbidity [2,3]. Therefore, postoperative neurological examination should be performed as early as possible; when the development of neurological deficits is detected, SEH should be considered and diagnosis, and possibly therapy, should start without delay.

## Case presentation

**Figure 1.**
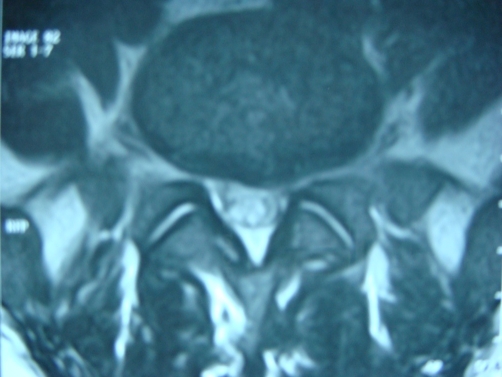
Preoperative axial MRI.

**Figure 2.**
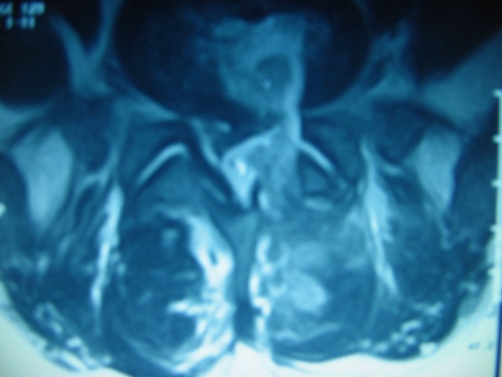
Postoperative SEH axial MRI.

**Figure 3.**
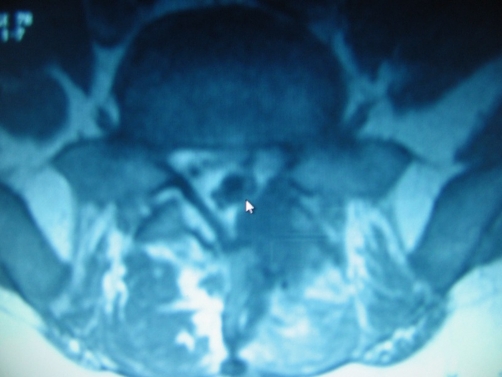
Axial MRI after SEH evacuation.

A 33-year-old Turkish woman patient was admitted with complaints of waist pain that extended down her left leg. She was diagnosed with left L5-S1 paramedian disc herniation and admitted for operation (Figure 1). In her preoperative neurological examination, she showed a left laseque of 45 degree +, hypoesthesia in left L5, S1 dermatomes and left foot dorsiflexion (DF) strength of 4/5. Left L5 hemipartial laminectomy, L5-S1 discectomy, and foraminotomy were performed. No complication occurred during the operation. Postoperative early neurological examination did not determine any new neurologic deficit. At the postoperative 12^th^ hour, neurological deficiency was observed. Upon neurological evaluation, left foot DF strength was 0/5, plantar flexion (PF) strength was 2/5, and urine incontinence and hypoesthesia in left L4, L5, S1 dermatomes were determined. Lumbar magnetic resonance imaging (MRI) confirmed acute SEH at the surgical site (Figure 2), and the patient underwent reoperation. Blood clots were evacuated and decompression was performed. There was no active bleeding at the site (Figure 3). Early clinical recovery was observed after reoperation, On postoperative 5^th^ day neurological examination showed left foot DF strength 2/5, PF strength 5/5 and there was no urine incontinence. The patient was admitted to physical therapy and a rehabilitation program on the 7^th^ day postoperation. 4 months postoperation, complete recovery was observed in the patient’s motor deficits.

## Discussion

SEH resulting in symptomatic spinal cord or cauda equina compression is one of the rare complications of spinal surgery. Although rare, symptomatic postoperative SEH have the potential for devastating neurological consequences requiring a spinal surgeon to be alert at all times for potential signs of SEH [2,14-17]. Retrospective series have estimated the incidence of symptomatic postoperative SEH to be 0.1% to 0.2% [1-4]. In contrast, asymptomatic MRI and computerized tomography (CT) studies have identified SEH in 33% to 100% of patients after lumbar disc or decompression surgery [5,8,18]. A current prospective study found that 58% of lumbar decompression surgery patients developed asymptomatic postoperative epidural hematoma of sufficient size to compress the thecal sac more than in the preoperative state. However, none of the patients developed new postoperative neurological deficits [14].

The risk factors for the development of postoperative SEH were determined as multilevel lumbar laminectomy procedures, preoperative coagulopathies, and vascular anomalies [1,3,10]. Multilevel laminectomies are more complex procedures that require exposure of larger epidural area. Complex spinal surgeries increase the risk for bleeding from the paravertebral muscles and contribute to the formation of SEH [3]. Groen and Ponssen [19] concluded that the etiology of a spontaneous SEH is a rupture of the internal vertebral venous plexus. Exposure of larger areas of the epidural space may increase the risk of insidious bleeding from the internal vertebral venous plexus and, subsequently, form a hematoma. Predisposing risk factors for SEH are the use of preoperative nonsteroidal anti-inflammatory drugs (NSAIDs), and intraoperative blood loss of more than 1 liter [2]. Moreover, patients over 60 years old, with Rh-positive blood types, intraoperative hemoglobin levels less than 10 g/dL, or an international normalized ratio (INR) greater than 2 within the first 48 postoperative hours have a statistically significantly increased risk [1]. There is no significant relationship between body mass index (BMI) and symptomatic SEH [3,14]. Similarly, there is no significant relationship between length of the operation and the volume of postoperative hematoma [14]. Preoperative durotomies are found to be insignificant factors in developing symptomatic hematoma [3,14]. However, multivariate analysis found that an age older than 60 years, multilevel procedures, and preoperative INR are associated with larger hematoma volumes [14]. Additionally, SEHs have been reported in those with liver and autoimmune diseases. SEHs have also been observed in individuals who undergo anticoagulant and thrombolytic therapies. Before the operation, special attention should be given to those patients with a known history of anticoagulant therapy or coagulopathy. However, coagulopathy is a risk even in those without a history [3,13]. However, in this study, we did not encounter coagulopathy in our preoperative and postoperative routine biochemical analysis and in our patient’s history.

In patients who require multilevel lumbar decompressions and those with preoperative coagulopathy, thorough hemostasis should be performed during the surgical procedure. SEH should always be kept in mind after spinal surgeries. Clinical evaluation is the most effective tool in the early diagnosis of SEHs. Detailed postoperative neurological examination is imperative and should be performed as soon as the patient is awake and cooperative. When postoperative neurological deterioration is detected in a patient, diagnostic studies, including MRI, to visualize possible epidural hematoma should be conducted, as it is imperative to diagnose an epidural hematoma as soon as possible to prevent the possible morbidity. After the diagnosis of postoperative SEH, early surgical exploration and evacuation of the hematoma should be performed [2,3].

In the literature, it is reported that patients who have undergone exploration and evacuation experienced the most extensive neurological recovery within 6 hours of diagnosis [2]. Patients who were surgically treated within 8 hours of diagnosis demonstrated less recovery [2,3]. In our case, as soon as SEH was detected and confirmed with lumbar MRI, surgical treatment was immediately applied. Epidural level blood clots were evacuated and active bleeding was not observed. The patient had normal urine incontinence on the first postoperative day and almost complete recovery of motor deficiency was achieved in the 4^th^ month.

## Conclusions

The incidence of epidural hematoma after spinal surgery is rare but a very serious complication. SEHs can cause significant spinal cord and cauda equina compression, requiring surgical intervention. Clinical evaluation is the most efficient tool in the early diagnosis of spinal epidural hematoma. Detailed postoperative examination should be performed as soon as the patient is awake and cooperative. Timely diagnosis and treatment of postoperative SEH are of the utmost importance. Prognosis of postoperative SEH depends on the development of symptoms, time of the surgery, level of spinal involvement, and degree of neurological deficit. The best results are achieved by early surgical intervention performed within 6 hours of symptom onset. In patients who underwent surgical intervention after 6 hours, less neurological recovery is achieved and morbidity increases. Once diagnosed, patients should immediately be taken into the operation room for emergency surgical exploration and evacuation of the hematoma.

## Abbreviations

BMI: Body mass index
CT: Computerized tomography
DF: Dorsiflexion
INR: International normalized ratio
MRI: Magnetic resonance imaging
NSAIDS: Non steroidal anti inflammatory drugs
PF: Plantar flexion
SEH: Spinal epidural hematoma
